# Clinically Explored Virus-Based Therapies for the Treatment of Recurrent High-Grade Glioma in Adults

**DOI:** 10.3390/biomedicines9020138

**Published:** 2021-02-01

**Authors:** Amanda V. Immidisetti, Chibueze D. Nwagwu, David C. Adamson, Nitesh V. Patel, Anne-Marie Carbonell

**Affiliations:** 1 Robert Wood Johnson Medical School, Rutgers University, New Brunswick, NJ 08901, USA; 2 School of Medicine, Emory University, Atlanta, GA 30322, USA; chibueze.dominic.nwagwu@emory.edu; 3 Department of Neurosurgery, School of Medicine, Emory University, Atlanta, GA 30322, USA; cory.adamson@emory.edu; 4 Atlanta VA Healthcare System, Decatur, GA 30033, USA; 5 Department of Neurosurgery, Robert Wood Johnson Medical School, Rutgers University, New Brunswick, NJ 08901, USA; patel236@njms.rutgers.edu; 6 OncoSynergy, Inc., Stamford, CT 06902, USA; anne-marie@oncosynergy.com

**Keywords:** glioblastoma, high-grade glioma, refractory glioma, virotherapy, oncolytic viruses, neuro-oncology, recurrent glioblastoma, chimeric viruses, clinical trials

## Abstract

As new treatment modalities are being explored in neuro-oncology, viruses are emerging as a promising class of therapeutics. Virotherapy consists of the introduction of either wild-type or engineered viruses to the site of disease, where they exert an antitumor effect. These viruses can either be non-lytic, in which case they are used to deliver gene therapy, or lytic, which induces tumor cell lysis and subsequent host immunologic response. Replication-competent viruses can then go on to further infect and lyse neighboring glioma cells. This treatment paradigm is being explored extensively in both preclinical and clinical studies for a variety of indications. Virus-based therapies are advantageous due to the natural susceptibility of glioma cells to viral infection, which improves therapeutic selectivity. Furthermore, lytic viruses expose glioma antigens to the host immune system and subsequently stimulate an immune response that specifically targets tumor cells. This review surveys the current landscape of oncolytic virotherapy clinical trials in high-grade glioma, summarizes preclinical experiences, identifies challenges associated with this modality across multiple trials, and highlights the potential to integrate this therapeutic strategy into promising combinatory approaches.

## 1. Introduction

### 1.1. Background

Primary brain tumors are classified by the World Health Organization (WHO) into four subgroups: grades I–IV. Of these, high-grade gliomas (HGGs), which include grades III and IV, are associated with high morbidity and mortality, highlighting the need for novel therapeutic approaches [1]. Glioblastoma (an HGG subset of WHO grade IV) continues to be one of the most formidable cancer diagnoses for several reasons. It is highly invasive, and its infiltrative growth pattern poses a challenge when attempting complete surgical resection. Even after tumor resection, parenchymal tissue surrounding the resection cavity is highly infiltrated with glioblastoma cells, facilitating the recurrence of disease. The current standard of care for glioblastoma includes maximally safe surgical resection, radiation, and chemotherapy [2]. Development of therapeutic resistance to standard chemotherapy is inevitable. Despite standard-of-care treatments, prognosis remains poor. The median survival for patients diagnosed with glioblastoma is 15 months, and the 2-year relative survival rate is 26% [3]. There is currently no standard of care for recurrent glioblastoma, which warrants the investigation of novel treatment strategies. Additionally, systemic delivery of therapeutics into the central nervous system (CNS) is hampered by the blood–brain barrier (BBB), which excludes many intravenously delivered agents from reaching effective concentrations in the brain. The integrity of the BBB is largely maintained by tight junctions between endothelial cells of cerebral capillaries. This barrier functions both to keep neuro-antigens out of systemic circulation, where they may be immunogenic, and to keep large molecules out of the brain, where they can cause toxicity or loss of function.

Methods to overcome this therapeutic challenge include modified direct delivery methods such as convection-enhanced delivery (CED), in which a specialized catheter is stereotactically placed into the targeted region of brain and therapeutics can be infused directly into parenchymal tissue [4]. Another direct delivery approach is to infuse therapeutics intra-arterially with an osmotic agent such as mannitol. This dehydrates endothelial cells and transiently disrupts tight junctions that form the BBB, thereby allowing drugs to enter the brain [5]. Additionally, a focused ultrasound can be used to enhance the delivery of systemically administered drugs that would otherwise be excluded by the BBB. In this approach, lipid-encased perfluorocarbons are administered intravenously. Under local stimulation with low-frequency ultrasound, these microbubbles oscillate and create mechanical forces that transiently and reversibly disrupt endothelial tight junctions, thereby allowing therapeutics to enter the brain [6]. These methods are warranted to reliably deliver a variety of therapeutics, including oncolytic viruses (OVs) into the CNS.

Finally, the immune-privileged status of the CNS is speculated to prevent robust activation of T lymphocytes, dampening the antineoplastic activity of the immune system. Due to these challenges, prognosis remains poor and there is an unmet need for additional therapies for glioblastoma. As additional therapeutic areas are explored, the use of OVs in glioblastoma shows promise and warrants further investigation.

### 1.2. Historical Context

**Figure 1:**
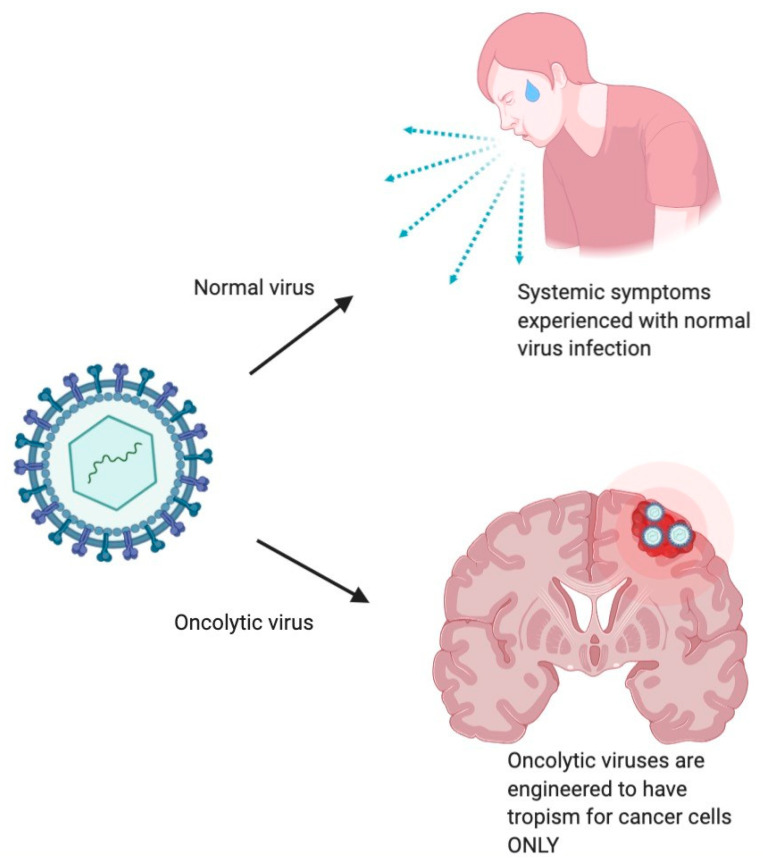
Oncolytic viruses have been engineered to exhibit selective tropism for cancer cells. Mechanistically, this entails that they rarely infect normal tissues, therefore reducing the systemic signs and symptoms that would be normally experienced with parental strains.

The utility of viruses to induce tumor cell death was initially observed by DePace in 1912 [7]. In this case report, a woman with cervical cancer sustained a dog bite and was treated with Pasteur’s attenuated rabies vaccine. Subsequently, regression of her cervical tumor was noted. This incidental finding prompted deeper inquiry into the use of viruses to treat solid tumors. The first preliminary clinical trial using an oncolytic virus to treat neoplasm was conducted when the rabies vaccine was given to 30 patients with melanomatosis, of which 8 showed regressive changes [8]. These early findings paved the way for more sophisticated oncolytic virotherapies using engineered viruses that exhibit selectivity tropism for cancer cells (Figure 1). Although there are many subtypes of oncolytic viruses, they can broadly be divided into replication-deficient or replication-competent viruses.

### 1.3. Mechanism of Antitumor Effect of Oncolytic Viruses

Replication-deficient viruses can be used functionally as viral vectors to deliver genes that, when expressed, cause tumor cell death and subsequent immune response (gene mediated cytotoxic immunotherapy).

**Figure 2:**
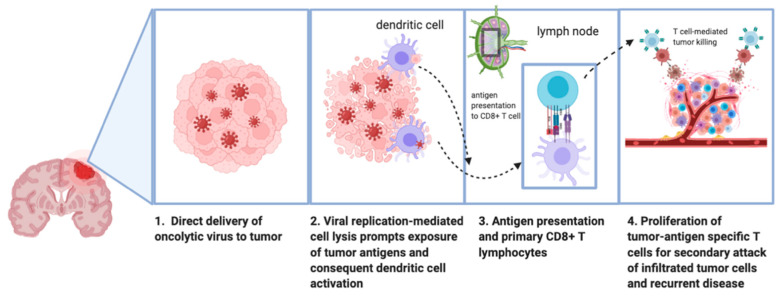
Mechanism of action of oncolytic viruses in the treatment of high-grade glioma.

In contrast, replication competent viruses selectively infect tumor cells and continue to replicate until the cell lyses. Their tendency to preferentially infect tumor cells is partially due to the loss of antiviral mechanisms in the malignant phenotype [9]. To fully characterize the implication of OVs, it is necessary to recognize the immunosuppressive nature of the tumor microenvironment prior to therapy, in which cytokines such as TGF-β, IL-10, and prostaglandin E are upregulated and effectively “mask” the tumor from the immune system [10]. Additionally, local regulatory T cells (T_reg_) and myeloid-derived suppressor cells (MDSCs) further dampen the immune response against tumor cells by preventing recruitment of T cells, B cells, and natural killer (NK) cells. OV therapy reverses this immunosuppressed tumor microenvironment to “unmask” the tumor from the immune system. The antitumor effect of a replication competent virus is two-fold: first, cell death of the infected cancer cell occurs with viral replication and lysis. After lysis, viral progeny continues to selectively infect neighboring tumor cells, and the cycle continues. There has been evidence to show that, when replication-competent oncolytic viruses are injected into a tumor, their antitumor effect can even be exerted on neighboring noninjected tumors [11,12] Second, OV therapy stimulates innate and adaptive immune responses against both viral and tumor antigens, as described by Gujar et al. Upon introduction of the OV, pathogen-associated molecular patterns (PAMPs) associated with the virus are recognized by pattern recognition receptors (PPRs) on cells of the innate immune system, including macrophages, monocytes, and dendritic cells. The antiviral pro-inflammatory cascade that follows includes the release of several cytokines including IFN-α, β, and λ; IL-1β; IL-6; IL-12; TNF-α; and granulocyte macrophage colony-stimulating factor (GM-CSF). Furthermore damage-associated molecular patterns (DAMPs) are also detected by PRRs. All of these events promote the presentation of antigens to CD4+ T cells in the lymph nodes, which drive the maturation of B and CD8+ T cells that carry out an adaptive immune response against tumor cells [13] (Figure 2).

Due to the ability of OVs to activate an antitumor immune response, they are attractive candidates for combination with immune checkpoint inhibition [10,14,15,16]. The most prominent checkpoint molecules are programmed cell death protein-1 (PD-1) and cytotoxic T-lymphocyte-associated protein 4 (CTLA-4). These are constitutively expressed on the surface of regulatory T cells and are upregulated on the surface of cytotoxic T cells during immune response. They serve to reduce apoptosis in regulatory T cells, consequently dampening the cytotoxic T lymphocyte-mediated immune response. The inhibition of these checkpoint molecules allows for a more robust cytotoxic T cell-mediated antitumor response to be elicited after OV therapy, a potential combinatory approach that is being explored clinically [17,18].

**Figure 3:**
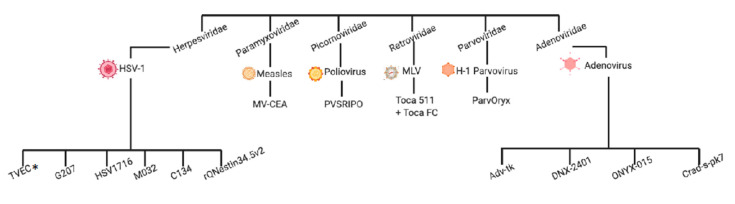
Family tree of oncolytic viruses explored in the high-grade glioma setting. * Talimogene Laherparepvec (TVEC) has not been used in this setting.

**Figure 4:**
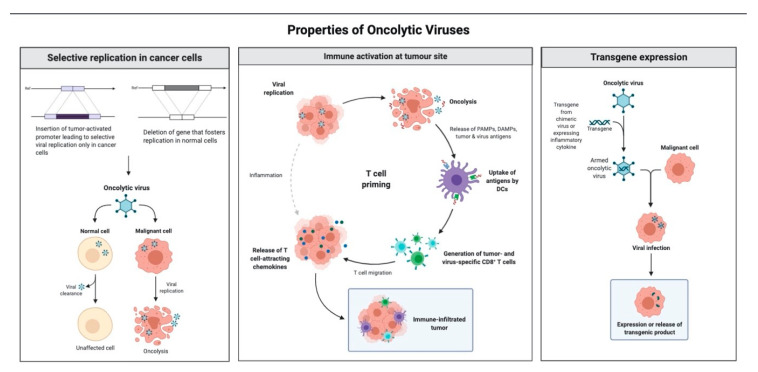
This depicts the various strategies adopted in the engineering of oncolytic viruses to grant selective tropism for malignant cells. The image was adapted from Groeneveldt et al., Trends Immunol 2020.

Today, a wide range of oncolytic viruses from multiple viral families are being explored clinically for the treatment of HGG and are the focus of this review (Figure 3). There are many OVs that have been studied preclinically in glioma models and reviewed extensively [19,20,21]. Viral therapies include those with modifications made to the *Herpesviridae, Adenoviridae, Paramyxoviridae*, and *Reoviridae* families as well as chimeric viruses that are engineered with transgenes to augment an antitumor effect (Figure 4): modification strategy deletion of neurovirulence genes, introduction of reporter genes, and interruption of viral genes necessary for replication in noncancerous cells. This review (1) surveys the current landscape of replication competent oncolytic virotherapy explored clinically in the treatment of HGG in adults, (2) provides clinicians with an adequate framework to assess the outcomes of clinically tested virotherapies, and (3) identifies future directions and potential areas of investigation in this emerging therapeutic field.

## 2. Clinical Experiences with Virotherapy in High-Grade Glioma

### 2.1. Herpesviridae

Multiple members of the herpes virus family, particularly, of the *herpes simplex virus-1* (HSV-1) subtype, have been modified and studied in clinical trials. *Herpesviridae* are double-stranded DNA viruses that are highly lytic, a property that renders them ideal for oncolytic virotherapy [9].

#### 2.1.1. Talimogene Laherparepvec (TVEC, OncoVex^GM-CSF^, or IMLYGIC)

In 2015, TVEC became the first United States Food and Drug Administration (FDA)-approved oncolytic virotherapy and was initially indicated for metastatic melanoma. TVEC is one of the most widely studied oncolytic viruses, with multiple clinical trials completed and in progress. TVEC was engineered by modifying the HSV-1 virus to improve replication competence, to decrease virulence, and to improve its profile as an oncolytic agent [9,10,11,12]. Notably, the addition of GM-CSF increased the immunogenicity of TVEC by attracting neutrophils to the site of viral infection and by stimulating stem cells to differentiate into granulocytes and monocytes, thereby augmenting the antitumor response [9]. 

To date, TVEC has not been used in the setting of glioblastoma. A recent search of the national clinical trials database using the terms “TVEC” and “cancer” revealed 21 active trials currently recruiting patients to continue testing this virotherapy in a variety of indications including melanoma, breast, pancreatic, liver, and colorectal cancers. TVEC is now tested in combination with other therapeutic agents, most notably, checkpoint inhibitors. There are currently 3 active trials recruiting participants to test TVEC in combination with pembrolizumab (KEYTRUDA), an anti-PD1 IgG4 (NCT03069378, NCT02965716, and NCT02509507) and 3 trials that are active but not yet recruiting (NCT02626000, NCT02263508, and NCT03842943). Of note, there are also 3 active trials currently recruiting participants to test TVEC in combination with nivolumab (Opdivo), another anti-PD1 IgG4 (NCT03597009, NCT02978625, and NCT03886311).

#### 2.1.2. HSV G207

Similar to TVEC, HSV G207 is also a modified HSV-1. It was also engineered to demonstrate decreased neurovirulence and improved safety profile through distinct modifications, which can be referenced in Markert et al. 2014. Of note, G207 has an *E. coli LacZ* gene insertion that interrupts the *UL39* gene, which codes for ICP6, a ribonucleotide reductase necessary for viral replication in nondividing cells. This interruption restricts G207 replication to activity to actively dividing cancer cells. Furthermore, the *E. Coli LacZ* acts as a reporter gene that can be tested using a histochemical assay, indicating whether viral replication was successful, which gives this therapy additional clinical utility [22,23,24]. Finally, G207 retained susceptibility to antiviral therapy, which can be initiated if the need to control viral replication arises [23]. 

The safety of G207 was demonstrated by a phase I trial (NCT00157703) and described by Markert et al. 2014. In this trial, 9 patients with recurrent malignant glioma underwent tumor biopsy followed by injection of G207 into 5 sites. Within the next 24 h, a single f 5 gray dose of radiation was administered in an effort to enhance viral replication. Six of the nine patients showed stable disease or partial response. Three patients showed radiographic response to treatment. Median survival (from time of G207 inoculation) was 7.5 months [23]. A phase Ib/II study (NCT00028158) was completed and described by Markert et al. 2000 [23]. In the phase Ib portion of the study, 21 patients were given intratumoral G207 and observed for safety. Four out of 21 patients remained alive at the time of submission, with a mean of 12.8 months post-inoculation (range 7–19 months). Mean survival time from inoculation to death of the remaining 17 patients was 6.2 months following inoculation (range 1–13 months). Mean survival from date of diagnosis for 13 glioblastoma patients was 15.9 months (range 12–22 months). There was no evidence of HSV encephalitis or toxicities exclusively attributed to the administration of G207.

Currently, there are two ongoing phase I clinical trials investigating the use of G207 when combined with a single dose of radiation in pediatric patients with recurrent supratentorial brain tumors (NCT02457845) and cerebellar brain tumors (NCT03911388). In these trials, G207 is infused intratumorally and is followed by a subtherapeutic 5 gray dose of radiation within 24 h of viral inoculation.

#### 2.1.3. HSV1716

Like G207, HSV1716 was also engineered to have reduced neurovirulence while maintaining the ability to replicate in actively dividing cells [25]. Unlike G207, HSV1718 retains the ability to replicate in nondividing cells. Although a disruption of this property may improve the safety profile of G207, it may account for the fact that HSV1716 demonstrates greater replication competence and can be administered at comparatively lower doses [26].

The safety of HSV1716 has been demonstrated in early clinical trials. In a phase I trial conducted in the United Kingdom, 9 patients were given intratumoral injections of HSV1716. Of these, 4 patients remained alive 12–24 months after treatment. There was no incidence of herpes-induced encephalitis or adverse events attributed to HSV1716 [26]. In a subsequent phase I study, 12 patients were given intratumoral HSV1716 followed by tumor resection [11]. In a third study, 12 patients underwent surgical resection of high-grade glioma, followed by injection of HSV1716 into the resection cavity in an effort to target residual tumor cells. Ten out of 12 patients were positive for HSV DNA in tumor tissue surrounding the injection site, and four were positive for HSV DNA in tissue that was spatially distinct from the original site of inoculation. At the time of publication, 3 patients remained alive at a range of 18–22 months [27].

There was recently a phase I trial (NCT02031965) initiated in which pediatric patients with refractory/recurrent HGG were to be HSV1716 peritumorally after maximal tumor resection. As of November 2016, VIRTTU Biologics reported that the trial was terminated due to a lack of recruitment.

#### 2.1.4. rQNestin34.5v.2

As previously discussed, although some gene modifications can improve the safety profile of oncolytic HSVs, engineering such viruses can hamper their replication competence in a clinical setting. To circumvent this, rQNestin34.5v.2 (herein rQNestin) was engineered to conditionally express replication competence in malignant glioma cells. Nestin, an intermediate filament, is a molecular marker of malignant glioma (expression was confirmed in 6 out of 6 human glioma lines and in 3 out of 4 primary glioma cells) [28]. This viral genome was engineered such that the gene that controls replication competence is located downstream from a synthetic Nestin promotor. Thus, rQNestin replication is impaired in cells that do not express Nestin, and robust replication is only seen in Nestin-expressing glioblastoma cells [28].

There is currently one active and recruiting phase I clinical trial in progress (NCT03152318) to evaluate rQNestin in 108 adults with recurrent malignant glioma. In arm A, a single dose of rQNestin is to be administered intratumorally in escalating doses until a maximum tolerated dose (MTD) or highest tolerated dose (HTD) has been established, at which point patients will be enrolled into arm B. Here, patients will receive pretreatment with cyclophosphamide (CPA), an immunomodulating agent, in a single IV infusion 2 days prior to one intratumoral dose of rQNestin. The rationale for the use of CPA is to dampen the host antiviral response that would limit the replication of rQNestin. In glioma rodent models, the addition of CPA to OV therapy was found to enhance viral replication and oncolysis and to prolong the expression of viral transgenes [29,30,31]. This effect is achieved through a CPA-mediated decrease in the expression of host antiviral cytokine mRNA [32].

#### 2.1.5. M032

M032 is another conditionally replication competent HSV-1 that is lytic in tumor cells. It is distinguished from other oncolytic viruses because it was engineered to express human IL-12 prior to host cell lysis, which stimulates an immune response against remaining tumor cells and propagates the antitumor effect of M032 [33,34]. Furthermore, IL-12 exerts an antiangiogenic effect, which may further contribute to the efficacy of M032 [35,36].

There is currently one active and recruiting phase I trial (NCT02062827) in which 36 adults with recurrent malignant glioma receive a single intratumoral dose of M032.

#### 2.1.6. C134

C134 is a second-generation chimeric oncolytic virus derived from ICP34.5-deleted HSV-1. ICP34.5 allows the wild-type virus to infect normal cells despite host antiviral defenses. Deletion of this gene reduces C134′s virulence and protein synthesis in normal cells. In an effort to restore viral synthesis in glioma cells, the *IRS1* gene from human cytomegalovirus (HCMV) was introduced into the genome. The *IRS1* gene product improves this virus’s oncolytic effect in glioma cells by allowing for late viral synthesis and does not restore wild-type neurovirulence in noncancerous cells [37,38,39]. These modifications improve both the safety profile and antitumor effect of C134 compared to both wild-type or ICP34.5-deleted HSV-1 [38,40].

There is currently one active phase I trial (NCT03657576) in which 24 adults with recurrent glioblastoma receive C134 inoculation at 1–5 sites within their tumor.

### 2.2. Adenoviridae

#### 2.2.1. Aglatimagene Besadenovec (AdV-tk)

This is a nonreplicating adenoviral vector modified to contain the herpes simplex thymidine kinase gene and can be administered in combination with valacyclovir to elicit an antitumor effect. When administered locally into the tumor bed following surgical resection, the viral vector infects remaining cancer cells and causes them to express the viral thymidine kinase gene. This is followed by oral administration of valacyclovir, an antiherpetic nucleoside analog. Thymidine kinase phosphorylates valacyclovir, which is incorporated into cancer cell DNA and inhibits further DNA synthesis or repair, causing cell death. This effect can be more pronounced when given in combination with radiation, as this causes strand breaks in glioma cell DNA, causing a greater degree of incorporation of phosphorylated valacyclovir, thereby inducing selective cell death. Following this, the adaptive immune system is triggered and immune effector cells amplify the antitumor effect [41].

In a phase II multicenter study described by Wheeler et al. (NCT00589875), 48 patients completed therapy with AdV-tk. Patients underwent surgical tumor resection, and AdV-tk was subsequently injected into 10 sites within the tumor bed. Valacyclovir was initiated 1–3 days after this, and radiation therapy was initiated 4–13 days post vector injection. Patients were also given temozolomide following injection of AdV-tk. No dose-limiting toxicities were observed, and the treatment group showed a 3.6 month increase in median OS [41].

#### 2.2.2. DNX-2401 (Tasadenoturev, Formerly Delta-24-RGD)

DNX-2401 is an oncolytic adenovirus engineered to selectively replicate in malignant cells. It was granted both fast track and orphan drug designation by the FDA. DNX-2401 was produced with two critical modifications: the first restricts its replication to malignant cells that display a dysfunctional retinoblastoma (Rb) pathway, which improves the safety profile of this virotherapy [42,43]. The second modification is an insertion of an Arg-Gly-Asp (RGD) peptide motif, which increases interactions with tumor integrins at the cell surface. This is thought to augment viral gene transfer and to increase the efficacy of DNX-2401 [44].

In a completed phase I study (NCT00805376), DNX-2401 was administered intratumorally to 37 patients with recurrent malignant glioma. In study arm A, 25 patients received a single dose of DNX-2401 intratumorally. In study arm B (treat-resect-treat), 12 patients received an intratumoral injection of DNX-2401 followed by tumor resection 14 days later with DNX-2401 injection into the resection cavity. Twenty percent of the patients in study arm A survived over 3 years after treatment, and 12% showed durable complete responses. An analysis of the post treatment samples from study arm B showed the immunogenic effect of DNX-2401, as there was evidence of viral replication and spread within the tumor following initial inoculation [45]. Following this, a phase 1b study (NCT02197169, TARGET-I) randomized 27 patients with recurrent glioblastoma to receive either DNX-2401 alone or with interferon-gamma (IFN). Notably, IFN was poorly tolerated and did not provide clinical benefit over DNX-2401 alone. Among both arms, OS-12 was 33% and OS-18 was 22%. Reported adverse events included fatigue, headache, and seizures consistent with existing disease [46]. Another phase I trial completed in Spain combined DNX-2401 with two 28 day cycles of temozolomide (TMZ) in patients with recurrent glioblastoma (NCT01956734) [47]. The preliminary results for this trial were presented at the American Association for Cancer Research annual meeting in 2017. At that time, 31 patients underwent tumor resection and intraparenchymal injection of DNX-2401 followed by 4 cycles of TMZ. The adverse events recorded were attributable to TMZ. Interestingly, seropositive patients who had neutralizing antibodies prior to treatment showed more favorable outcomes. 

A recently completed phase 2 trial (NCT02798406, CAPTIVE/KEYNOTE-192) combined DNX-2401 with pembrolizumab (KEYTRUDA) in 49 patients with recurrent glioblastoma. The results were presented at the 2020 Society of Neuro-Oncology annual meeting and demonstrated this combination to be safe. The most commonly reported treatment-related adverse events included headache, brain edema, and fatigue. Efficacy endpoints included mOS (12.5 months), OS-12 (54.5%), and OS-18 (20.8) [48]. A phase 3 trial is planned but not yet registered through the national clinical trials database.

There is currently one active clinical trial using DNX-2401 in the setting of high-grade glioma. This phase I trial (NCT03896568) will enroll 36 patients with recurrent high-grade glioma. In this treat-resect-treat design, patients will receive bone-marrow-derived human mesenchymal stem cells (BM-hMSCs) loaded with DNX-2401 through arterial injection. After 2 weeks, patients will undergo tumor resection and receive another course of BM-hMSCs loaded with DNX-2401.

#### 2.2.3. ONYX-015

Similarly to DNX-2401, ONYX-015 is a selectively replication competent adenovirus. In this case, it was engineered with a gene deletion in the *E1B* region. In the wild-type virus, the *E1B* gene product allows the virus to counteract the host P53 pathway. When this is disrupted, the virus is unable to replicate in cells with a functional P53 pathway but is replication competent where it is dysregulated [49,50]. In an analysis of 206 glioblastoma tumors from the Cancer Genome Atlas (TCGA), approximately 85% was found to have dysregulations in the P53 pathway [51].

ONYX-015 has been used in a range of trials, demonstrating its safety, most notably in a phase I dose escalation trial in recurrent malignant glioma [52]. In this study, 24 patients were enrolled (6 per dosing cohort) and underwent tumor resection that was immediately followed by injection of ONYX-015 into 10 sites within the tumor resection cavity. There were no serious adverse events reported that could be definitively attributed to ONYX-015 administration. Although administering the virus at the time of tumor resection was shown to be safe, there was no definite antitumor activity. The median time to progression was 46 days (range 13–452 days), and median survival was 6.2 months (range 1.2–28 months).

#### 2.2.4. CRAd-S-pk7

A newly emerging approach to delivering virotherapy involves loading neural stem cells with an oncolytic adenovirus that is delivered locally. CRAd-S-pk7 is a conditionally replicating adenoviral vector. “S-pk7” refers to the addition of a survivin promotor and a polycysteine, which together enhance tumor specific viral replication and improve transduction efficacy of the viral vector [53,54]. Neural stem cells are used as a delivery mechanism for virotherapy due to their tendency to migrate towards neoplastic tissue and to aid in more direct delivery of therapeutics [55].

There is currently one active clinical trial in which neural stem cells loaded with CRAd-S-pk7 will be administered to 36 patients with recurrent malignant glioma in a phase 1 study (NCT03072134). In the first study arm, patients with unresectable tumors will undergo a biopsy followed by injection of NSC loaded with CRAd-S-pk7 into the tumor. In the second study arm, patients with resectable tumors will undergo tumor resection followed by injection of neural stem cells loaded with CRAd-S-pk7 into the resection cavity. Following injection, both arms will receive standard-of-care chemoradiation. Tumor response is to be assessed on MRI.

### 2.3. Retroviridae

#### Vocimagene Amiretrorepvec + (5-fluorocytosine(6-amino-5fluoro-1H-pyrimidin-2-one)) − (Toca 511 + Toca FC)

This is a dual agent combination. The first agent (Vocimagene amiretrorepvec or Toca 511) is a modified, nonlytic retroviral vector engineered from the murine leukemia virus (MLV) to include the yeast cytosine deaminase (CD) gene [56]. Toca 511 selectively infects cancer cells, causing the CD gene to be integrated into the genome of actively dividing cells [33,56]. Subsequently, Toca FC (a prodrug) is given orally and is converted to 5-fluorouracil (5-FU) in cells expressing CD. 5-FU, a pyrimidine analog, is an antimetabolite that is used widely in the treatment of other malignancies (particularly of the breast and gastrointestinal tract). Active metabolites of 5-FU inhibit thymidylate synthase and disrupt nucleic acid synthesis. Collectively, the downstream effects of 5-FU result in DNA strand breaks and death of actively diving cells [57]. Preclinical models have also shown that 5-FU may also induce death in neighboring MDSCs (which are implicated in the immunosuppressed nature of the tumor microenvironment) and further stimulate the body’s immune response [58]. Additionally, 5-FU was shown to be a radiosensitizing agent both in vivo and in vitro when tested in radioresistant glioma cell lines, which highlights the potential for concomitant radiotherapy as a possible therapeutic combination [59].

Clinical trials using the Toca 511 + Toca FC combination were conducted under breakthrough designation awarded by the US FDA. In an initial phase I trial (NCT01470794), 58 patients with recurrent HGG underwent tumor resection followed by Toca 511 injection into the resection cavity and Toca FC dosing throughout the course of the 30 week study. Following this, two separate cohorts also received bevacizumab or lomustine. Preliminary results from 45 patients showed the overall survival in patients with HGG to be 13.6 months and in glioblastoma to be 11.6 months [60]. In a post hoc analysis of 56 enrolled patients (53 of whom were evaluable), the objective response rate was found to be 11.3% and mOS was 11.9 months. At the time of study conclusion, all 6 responders remained alive and in complete remission 33.9 to 52.5 months after treatment initiation [61]. In another phase I study (NCT01156584), 54 patients with recurrent HGG were recruited into cohorts that received one of the following interventions: (1) intratumoral injection of Toca 511, (2) IV injection of Toca 511 daily for 3 days, or (3) IV injection of Toca 511 daily for 5 days. All patients subsequently received oral Toca FC. In a third phase I study (NCT01985256), 17 patients were given an IV bolus of Toca 511. After 11 days, patients underwent surgical resection and intracranial injection of Toca 511 into the resection cavity followed by Toca FC.

An ongoing trial using Toca 511 and Toca FC was recently discontinued by the sponsor. Dubbed “Toca 5,” NCT02414165 was a randomized phase II/III trial in which the Toca 511 and Toca FC combination was tested against a standard-of-care control arm in patients with recurrent glioblastoma or anaplastic astrocytoma. This study enrolled 403 patients (201 were randomized into the experimental arm, and 202 were in the control arm). The experimental arm intervention included injection of Toca 511 into the resection cavity at the time of surgery followed by oral Toca FC six weeks later. In patients who underwent treatment, the Toca 511/FC combination did not demonstrate efficacy, as there was no improvement in overall survival [62].

### 2.4. Picornoviridae

#### PVSRIPO

PVSRIPO is a replication-competent recombinant poliovirus in which the internal ribosome entry site (IRES) is replaced with that of human rhinovirus, effectively abolishing neurovirulence in nonmalignant cells [63]. Poliovirus recognizes and binds to CD155, a tumor antigen that is widely expressed in solid tumors. Cytotoxic replication of PVSRIPO initiates malignant cell death, generates inflammation, and primes the immune system to recognize tumor cells [64,65].

In a phase I trial (NCT01491893), 61 adult patients with recurrent grade IV HGG were treated with 1 intratumoral infusion of PVSRIPO via CED at 7 escalating dose levels. Patients were given a booster of the poliovirus immunization 2 weeks prior to infusion. An overall survival of 21% was observed at 24 months and was sustained at 36 months. The median overall survival was 12.5 months [66].

There are currently three clinical trials investigating treatment of HGG with PVSRIPO. A phase Ib trial (NCT03043391) is enrolling 12 pediatric patients with malignant glioma who will receive one intratumoral infusion of PVSRIPO and will be monitored for one year after treatment. A phase 1b/II trail (NCT03973879) plans to enroll 31 adults with recurrent grade IV glioma to receive intratumoral infusion of PVSRIPO followed by atezolizumab, a humanized IgG monoclonal antibody against PD-L1. Following this, tumor resection was planned at the discretion of the investigator. This trial was withdrawn, but the trial registration notes that resubmission is expected. In an ongoing phase II trial (NCT02986178), 122 adults with recurrent malignant glioma will receive intratumoral infusion of PVSRIPO alone or in combination with lomustine.

### 2.5. Reoviridae

#### Pelareorep (REOLYSIN)

REOLYSIN is an unmodified wild-type serotype 3 reovirus (respiratory enteric orphan virus) that is nonpathogenic in humans. It was found to have potential for use as oncolytic therapy due to increased replication in cells with upregulated *Ras* signaling, which is common in malignant cells [67,68,69].

In a phase I dose escalation trial, 12 patients with recurrent malignant glioma were given a single intratumoral stereotactic injection of REOLYSIN. One patient was noted to have stable disease, 10 had progressive disease, and one patient was not able to undergo further evaluation. The median overall survival was 21 weeks (range was 6–234 weeks), the median time to progression was 4.3 weeks (range 2.6–39), and a maximum tolerated dose was not reached [70]. Of note, viral shedding was noted in the saliva of one patient and in the feces of two. One patient was also positive for reovirus at the start of the study but became negative after treatment [70]. This warrants further investigation into the potential for maintenance of a viral reservoir and subsequent shedding when administering virotherapies. In another phase I dose escalation trial (NCT00528684), 15 adults with recurrent malignant gliomas were given REOLYSIN via CED in a single intratumoral injection over 72 h. Following this, 10 patients had stable disease, one had partial response, and four had progressive disease. The median overall survival was 140 days, and the median time to progression was 61 days [71]. This trial was the first in which an oncolytic virus was administered via CED in the United States.

There is currently one active phase I trial in which 6 pediatric patients with recurrent glioma will be enrolled. Patients will receive sargramostim (GM-CSF), on days 1 and 2, which is to be followed by a 60 min IV infusion of REOLYSIN on days 3–5. The combination of GM-CSF and REOLYSIN is expected to enhance immunogenicity through stimulation of dendritic cell maturation and increased presentation of tumor antigens [72]. This treatment will be repeated every 28 days for 12 cycles.

### 2.6. Paramyxoviridae

#### MV-CEA

MV-CEA is an oncolytic measles virus derived from the Edmonston vaccine lineage and has been shown to have antitumor effect against malignant glioma [73]. In this case, the measles virus is engineered to include human carcinoembryogenic antigen (CEA), which is a peptide marker that can be used to detect viral gene expression [74]. A toxicology study was completed to demonstrate safety in nonhuman primates to support a phase I/II clinical trial in recurrent glioma [74].

### 2.7. Parvoviridae

#### Parvovirus H-1 (H-1PV, Parv-Oryx)

Parv-Oryx is an oncolytic single-stranded DNA virus in which the natural host is the rodent. Parv-Oryx retains the ability to infect and replicate the inside of human cells but is not associated with pathology in nonneoplastic tissue [75,76,77,78]. Of note, its oncolytic mechanism of action is thought to work through the cathepsin-mediated cell death pathway, so it may be an effective therapeutic approach by which to target glioma cells with defective apoptotic pathways [79]. Parv-Oryx is also unique in that it readily crosses the blood–brain barrier, which is a clinically valuable feature as this has potential for intravenous administration and may circumvent the need for surgical catheter placement or intracranial injection if administered prior to tumor resection [80,81].

A phase I/IIa study of Parv-Oryx was completed in 2015 in patients with progressive primary or recurrent glioblastoma in Germany (NCT01301430) [80]. A total of 18 patients were enrolled equally into two study arms. In the first group, patients received ParvOryx in a treat-resect-treat study design. The virus was initially administered via intratumoral injection followed by resection, and another round of intracranial injection was administered into the tumor bed 9 days later. In the second study arm, the first treatment was given intravenously. Following this, treatment was similar to the first study arm (resection and intracranial injection 9 days later). Notably, clinical response was not found to be dependent on either the dose or route of entry, indicating that oncolytic parvovirus is able to cross the blood–brain barrier. This was further evidenced by the detection of viral RNA transcripts in tumor tissue collected from patients in the IV dose group. Resected tumor tissue from five patients showed CD4+ and CD8+ infiltration that was not present in historical controls and overlapped with viral RNA detection. These findings support antitumor immunogenicity in a subset of patients. The median overall survival was 464 days, and the median progression-free survival was 111 days [81].

### 2.8. Summary of Clinical Experiences Using OVs in High-Grade Glioma

As discussed, a variety of OVs have been used in clinical trials in patients with HGG. The completed clinical trials are summarized in Table 1. Trials that are in progress at the time of submission are summarized in Table 2.

## 3. Summary of Preclinical Experiences with Combinatory Virotherapy in Glioblastoma

Several combinatory strategies have been explored to render virotherapy more efficacious, with little to no neurological adverse effects in GBM mouse models.

PD-1 and CTLA-4 have been successfully targeted in other indications without much success in the glioma setting. Compounding evidence from a stream of recent publications attributed this phenomenon to the scheduling of checkpoint inhibition around surgery and possibly the molecular profile of patients [82,83,84]. Cloughesy et al. showed that administering neoadjuvant/adjuvant anti-PD-1 before and after surgical resection showed clinical benefit. This regimen was also shown to induce immune cell infiltration and augmented T cell receptor clonal diversity among tumor-infiltrating T lymphocytes [84].

Given these first successful instances of targeting PD-1 in glioma patients, the synergistic implications of checkpoint inhibition and augmented virotherapy-mediated immune response are active areas of preclinical investigation. Hardcastle et al. showed that oncolytic measles virus infection in vitro induced the secretion of DAMPs and upregulated PD-L1 expression [85]. This synergistic potential was further corroborated in vivo, where oncolytic measles virus combination with PD-L1 blockade was shown to significantly improve survival in a syngeneic glioblastoma model [85]. Similarly, Errington-Mais et al. recently demonstrated that viruses could prime the glioma microenvironment for ensuing checkpoint blockade [86]. Intravenously delivered reovirus upregulated tumor PD-L1 expression, thereby further opsonizing the tumor for subsequent anti-PD-L1 action. This combination ultimately led to improved survival in a preclinical mouse model of glioma [86]. The utility of virotherapy in combination with checkpoint blockade has also been proven to spur a potent secondary adaptive response. An anti-PD-1-expressing HSV, NG34scFvPD-1, was shown to improve survival in syngeneic immunocompetent glioblastoma mouse models [87]. Spectacularly, a second challenge with glioblastoma cells in mice already treated with the anti-PD-1 expressing oHSV proved futile, hence suggestive a vaccinal effect [87]. It has been well-characterized that PD-1 blockade leads to the consequent upregulation of its counterpart checkpoint molecule, CTLA-4 [84]. Saha et al. combined an IL-12-expressing HSV with anti-CTLA-4 and anti-PD-1 in a mouse glioma model [88]. This triple combination extended survival, increased T effector to T regulatory cell ratios, and led to subsequent rejection of glioblastoma re-challenge in the immunocompetent mouse model [88].

The extracellular matrix (ECM) has also been implicated in the propagation of phenotypes associated with the several hallmarks of cancer such as migration, immunosuppression, and therapeutic resistance [89]. Particularly, the desmoplastic state that is characteristic of most solid tumors is largely due to the increased aggregation and dysregulated organization of ECM proteins [89,90]. Moreover, the previously discussed oncolytic HSV variant, HSV1716, has been associated with altering high-grade glioma cytoskeletal dynamics, hence proving replication is ECM-dependent [91]. ECM proteins have, therefore, also become attractive targets for enhancing viral replication. Particularly, integrins have been shown to be upregulated in the glioma microenvironment. The integrin ligand-containing adenovirus, DNX-2401, demonstrated glioma cell lysis and subsequent release of DAMPs to elicit Th1 immune response in the immunocompetent glioma mouse model [92]. Integrin-mediated entry has also been utilized to improve both viral tropism and replication [93,94]. Lee et al. showed that the β1 integrin blockade improved replication of an HSV variant and promoted antitumor efficacy in patient-derived primary glioblastoma-bearing mice [95].

Efforts have been made to target replication-incompetency at the transcriptome level. Chemoradiation, which is part of the standard of care for HGG, exerts its effect by targeting the excessively replicative nature of glioma DNA. Chemoradiation and virotherapy have thus been explored preclinically as combinatory options. The standard HGG chemotherapeutic TMZ is an alkylating agent that delivers a methyl group to purine bases of DNA, consequently spurring DNA damage. GuhaSarkar et al. showed that TMZ administered post adenovirus + interferon-beta therapy resulted in a significant survival benefit compared to both modalities alone [96]. Since TMZ is typically given 4 weeks after surgery in the clinical setting, virotherapy could serve as an option to sensitize infiltrative glioma cells to the effects of chemoradiation.

## 4. Discussion

Inflammation poses a considerable challenge when designing clinical trials in the neuro-oncology space. Data assessing inflammatory responses from virotherapy clinical trials in patients with HGG are not always consistent, as this patient population tends to be immunosuppressed at baseline to varying degrees. Furthermore, standard-of-care chemoradiation and routinely prescribed steroid therapy to control tumor-associated cerebral edema can also be immunosuppressive. As noted in a clinical trial of HSV-1716, a patient who was on a high dose of dexamethasone at the time of viral administration had no resulting immune response [27]. 

Surgical intervention is also associated with some degree of inflammation, which may be addressed by modifying the delivery schedule. Administration of virotherapy at the time of resection, when there is additional inflammation at the site of disease, may poise the virus to be quickly neutralized by the immune system. Harrow et al., 2004, noted that HSV-1716 may have failed to produce lytic infection in the setting of surgery and proposed conducting viral inoculation at a time distinct from tumor resection [27]. To further minimize swelling, it may be prudent to collect pre-infusion biopsies greater than 48 h prior to the delivery of virotherapy so that the peak of swelling will have already elapsed at the time of viral inoculation. Another solution may be to administer virotherapy through implantable CED catheters, which can be left in place post-resection until surgery-associated inflammation has subsided and then used to infuse virotherapy into the resection cavity. In some trials using intracranial injection, up to 40 separate injections were proposed, which involves the placement and positional adjustment of multiple needles. The use of methods such as CED or intraarterial infusion can reduce the inflammation associated with multiple needle punctures. Finally, it is prudent to consider that an increase in the number of cells that are turning over during surgery may result in a loss of selectivity, as the virus may infect nonmalignant cells that would have otherwise been spared from infection [22]. 

The administration of a virus directly into the brain requires vigilance during adverse event monitoring due to the possibility of developing viral encephalitis. Although multiple clinical trials have demonstrated the safety of local administration of many OVs, it is important to consider modifications that can be made to engineered viruses to reduce neurovirulence in nonmalignant cells. In the event that viral encephalitis does develop, a lack of antiviral choice poses a significant challenge. Another risk associated with virotherapy is the potential for environmental shedding of the virus and subsequent infection of others. Forsyth et al., 2008, found that, after administering REOLYSIN, one patient was found to shed the virus in saliva and 2 were found to shed it in stool. It may be advisable to collect both stool and saliva samples to assess for environmental shedding when designing future virotherapy clinical trials. When assessing the degree of viral replication, a lack of assay specificity can also pose a monitoring challenge. Unless a distinct reporter gene is introduced to the viral genome, existing assays may not be able to differentiate between the wild-type virus and the engineered (therapeutic) virus. Harrow et al., 2004, noted this as a study limitation, as PCR could not distinguish between wild-type HSV from engineered HSV-1716 [27]. With regard to engineering modifications, although certain gene deletions may reduce neurovirulence, they can subsequently compromise the efficacy of the OV. For this reason, G207, which was engineered with an additional modification to improve the safety profile, exhibits a lower transduction efficacy than HSV-1716 [26]. Such a compromise may allow for the virus to be more readily neutralized by the immune system.

There are some aspects of virotherapy in neuro-oncology that are incompletely understood and warrant further study. Perhaps the most perplexing data across multiple virotherapy trials are the clinical outcome of patients in the context of their baseline serology. There are conflicting results, as they are likely dependent on both the patient characteristics and oncolytic virus in question. Markert et al., 2014, found the most significant responses to G207 (HSV) to be in seronegative patients but noted that the previous G207 trial found the best responses to be in seropositive patients [23]. Hu et al., 2006, found that seronegative patients who were given OncoVex GM-CSF (also an HSV) had more pronounced constitutional symptoms, which limited the maximum tolerated dose [12]. Patients were also found to seroconvert after viral administration; however, the data are again inconsistent. Chiocca et al. (2004) found that two out of 24 patients given ONYX-015 (an adenovirus) seroconverted from negative to positive [52]. Markert et al., 2000, found that only one patient from the highest dose level of G207 (HSV) seroconverted from negative to positive [22]. It may be beneficial to seroconvert patients using similar viral vaccines (when available) prior to initiating virotherapy. In a phase I trial of PVSRIPO (NCT01491893), patients were given the poliovirus booster 2 weeks prior to infusion. This may have primed the immune system for virotherapy and may have dampened the adverse events, allowing for a higher maximum tolerated dose. This warrants further investigation, as conclusive results are not seen across studies that have collected serological results. Of note, Hu et al. proposed a multi-dosing scheme in which patients were initially given a low dose of the virus to allow for seroconversion prior to administration of the intended dose, which may modulate the side effects without having a negative effect on tumor necrosis level [12]. As noted by Markert et al., 2014, to fully understand this phenomenon, a larger prospective study should be conducted to determine whether pretreatment exposure is a biomarker for response to virotherapy treatment [23]. This could aid in identifying certain populations of patients with HGG that may be better candidates for virotherapy and that may stand to gain greater clinical benefit from these therapies. 

As clinical trials advance to later phases, strategic combinations can augment the efficacy of single therapies. Markert et al., 2014, notably gave a subtherapeutic dose of concomitant radiation because it was thought to enhance viral replication [23]. Takahashi et al. showed the sensitization of previously radioresistant glioma cells after inoculation with a retrovirus in both in vitro and in vivo preclinical models [59]. Radiotherapy following treatment with Toca 511 and Toca FC is thought to exert a local effect on cells containing 5-FU, thereby sparing surrounding nonmalignant tissue [59]. 

The outlined body of work in the clinical exploration of OVs to date paves the way to continue to actively investigate their potential for the treatment of HGG. We expect future efforts to characterize this therapeutic approach as having clinical significance, particularly as part of a combinatory regimen. 

## Figures and Tables

**Table 1:** Completed clinical trials using oncolytic virotherapy in high-grade glioma.

Agent	NCT	Study Phase	Published Results	*n*	Study Population	Outcomes
G207	NCT00157703	Phase I	Markert et al. 2014	9	Recurrent malignant glioma	Safety demonstrated (AEs) Median survival from inoculation = 7.5 months mPFS = 2.5 months
	NCT00028158	Phase Ib/II	Markert et al. 2000	21	Recurrent malignant glioma	Safety demonstrated (AEs) Mean TTP = 3.5 months Mean OS = 15.9 (glioblastoma) and 40.5 (anaplastic astrocytoma)
HSV1716	(UK)	Phase I	Rampling et al. 2000	9	Recurrent malignant glioma	Safety demonstrated (AEs)
	(UK)	Phase I	Papanastassiou et al. 2002	12	Malignant glioma	Safety demonstrated (AEs)
	(UK)	Phase I	Harrow et al. 2004	12	Recurrent or newly diagnosed high grade glioma	Safety demonstrated (AEs)
AdV-tk	NCT00589875	Phase II	Wheeler et al. 2016	48	Newly diagnosed glioblastoma	Safety demonstrated (AEs, DLTs) mOS = 17.1 months mPFS = 8.1 months Surival at 1, 2, 3 years = 67%, 35%, 19%
DNX-2401	NCT00805376	Phase I	Lang et al. 2018	37	Recurrent malignant glioma	Safety demonstrated (AEs, DLTs) Study arm A (single injection)- Tumor reduction in 72% of patients mOS = 9.5 months Study arm B (infusion and resection) mOS = 13 months
	NCT02197169 (TARGET-1)	Phase Ib	Lang et al. 2017	27	Recurrent glioblastoma	Tolerability of DNX-2401 as monotherapy (compared to combination with IFN-gamma) demonstrated (AEs) OS-12 (33%) OS-18 (22%)
	NCT01956734 (Spain)	Phase I	Alonso et al. 2017	31	Glioblastoma at first recurrence	Safety demonstrated when combined with TMZ (AEs), efficacy endpoints not yet reported
	NCT02798406 (CAPTIVE/KEYNOTE-192)	Phase II	Zadeh et al. 2020	49	Recurrent glioblastoma	Safety demonstrated when combined with pembrolizumab (AEs) mOS = 12.5 months OS12 = 54.5%, OS18 = 20.8%
ONYX-015	-	Phase I	Chiocca et al. 2004	24	Recurrent malignant glioma	Safety demonstrated (AEs, DLTs) Median survival = 6.2 months (4.9 months for glioblastoma patients, 11.4 in AA/AO)
Toca511 + TocaFC	NCT01470794	Phase I	Cloughesy et al. 2016	43	Recurrent high grade glioma	Safety (AEs, DLTs) OS (HGG) = 13.6 months OS (glioblastoma) = 11.6 months For all evaluable patients: OS6 (87.9%), OS9 (72.4%), OS12 (52.5%), OS24 (29.1%) PFS = 3.2 months, PFS6 = 16.3%
	NCT01156584	Phase I	-	54	Recurrent high grade glioma	-
	NCT01985256	Phase I	-	17	Recurrent or progressive high grade glioma	-
	NCT02414165 (Toca 5)	Phase II/III	Cloughesy et al. 2020	201	Recurrent glioblastoma/anaplastic astrocytoma	Safety (AEs) mOS = 11.1 months Efficacy was not demonstrated over control arm
PVSRIPO	NCT01491893	Phase I	Desjardins et al. 2018	61	Recurrent glioblastoma	mOS = 12.5 months OS 24 M and 36 M = 21%
REOLYSIN	NCT00528684	Phase I/II	Forsyth et al. 2008	12	Recurrent malignant glioma	mOS = 21 weeks (range 6–234) mTTP 4.3 weeks (range 2.6–39) MTD not reached
	NCT00528684	Phase I/II	Kickielinski et al. 2014	15	Recurrent malignant glioma	mOS = 140 days mTTP = 61 days

Abbreviations: AE = adverse events; DLT = dose limiting toxicity; OS = overall survival; mOS = median overall survival; mTTP = median time to progression; mPFS = median progression free survival; PFS6 = progression free survival at 6 months; AA = anaplastic astrocytoma; AO = anaplastic oligodendroglioma; TMZ = temozolomide.

**Table 2:** Clinical trials in progress or results not yet reported using oncolytic viruses in high-grade glioma.

Agent	NCT	Study Phase	*n*	Trial Design/Population	Outcomes (Safety, Efficacy)
G207	NCT02457845	Phase I	12	Pediatric progressive or recurrent supratentorial tumors	Safety, tolerability (AEs) PFS, OS
	NCT03911388	Phase I	15	Pediatric recurrent or refractory cerebellar tumors	Safety, tolerability (AEs) PFS, OS
HSV1716	NCT02031965	Phase I Terminated by sponsor	2	Pediatric refractory/recurrent high grade glioma	MTD, PFS, and OS up to 15 years
rQNestin	NCT03152318	Phase I	108	Malignant glioma	MTD
M032	NCT02062827	Phase I	36	Recurrent malignant glioma	MTD TTP and survival up to 12 months
C134	NCT03657576	Phase I	24	Recurrent glioblastoma	Safety, tolerability (AEs) PFS- 3 d, 28 d, 3 M, 6 M, 12 M, OS up to 12 M
DNX-2401	NCT03896568	Phase I	36	Recurrent high-grade glioma	MTD, AEs Tumor response, TTP for 1 year
CRAd-S-pk7	NCT03072134	Phase I	12	Newly diagnosed malignant glioma	Neurological side effects, MRIs for progression
Toca 511 + Toca FC	NCT02598011 (Toca 7)	Phase Ib Terminated by sponsor	18	Newly diagnosed high grade glioma	DLTs
PVSRIPO	NCT03043391	Phase Ib	12	Pediatric recurrent malignant glioma	Toxicity, 24 month OS
PVSRIPO + atezolizumab	NCT03973879	Phase Ib/2 Withdrawn, resubmission expected	_	Recurrent malignant glioma	Safety (AEs), survival at 24 M
PVSRIPO + lomustine	NCT02986178	Phase II	122	Recurrent malignant glioma	Objective response (iRANO) at 24 and 36 M, duration of ORR, OS at 24 and 36 M, safety (AEs)
REOLYSIN + GM-CSF	NCT02444546	Phase I	6	Pediatric relapsed/refractory brain tumors	MTD (DLT), AE, mOS, OR, TTP

Abbreviations: AEs = adverse events; PFS = progression free survival; mPFS = median progression free survival; OS = overall survival; mOS = median overall survival; AEs = adverse events; MTD = maximum tolerated dose; DLTs = dose limiting toxicity; ORR = objective response rate; iRANO = immunotherapy response assessment in neuro-oncology; TTP = time to progression.

## Data Availability

No new data were created or analyzed in this study. Data sharing is not applicable to this article.

## Abbreviations

WHO	World Health Organization
HGG	high-grade glioma
CNS	central nervous system
BBB	blood–brain barrier
OV	oncolytic virus
T_reg_	regulatory T cell
MDSC	myeloid-derived suppressor cell
NK	natural killer
PAMP	pathogen-associated molecular pattern
PRR	pattern recognition receptor
DAMP	damage-associated molecular patterns
GM-CSF	granulocyte macrophage colony-stimulating factor
CTLA-4	cytotoxic T-lymphocyte associated protein 4
PD-1	programmed cell death protein-1
HSV	herpes simplex virus
FDA	Food and Drug Administration (of the United States)
CPA	cyclophosphamide
TMZ	temozolomide
CD	cytosine deaminase
5-FU	5-fluorouracil
CEA	carcinoembryogenic antigen
ECM	extracellular matrix
